# A Highly Glucose Tolerant ß-Glucosidase from *Malbranchea pulchella* (*Mp*Bg3) Enables Cellulose Saccharification

**DOI:** 10.1038/s41598-020-63972-y

**Published:** 2020-04-24

**Authors:** Lummy Maria Oliveira Monteiro, Ana Claudia Vici, Matheus Pinto Pinheiro, Paulo Ricardo Heinen, Arthur Henrique Cavalcante de Oliveira, Richard John Ward, Rolf Alexander Prade, Marcos S. Buckeridge, Maria de Lourdes Teixeira de Moraes Polizeli

**Affiliations:** 1 Faculdade de Medicina de Ribeirão Preto. Universidade de São Paulo. Bandeirantes Av., 3.900, 14049-900 Ribeirão Preto, SP Brazil; 2 Faculdade de Filosofia, Ciências e Letras de Ribeirão Preto. Universidade de São Paulo. Bandeirantes Av., 3.900, 14040-901 Ribeirão Preto, SP Brazil; 3 Laboratório Nacional de Biociência (LNBio), Centro Nacional de Pesquisa em Energia e Materiais (CNPEM), Campinas, SP Brazil; 4 Department of Microbiology and Molecular Genetics. Oklahoma State University, Stillwater, USA; 5 Instituto de Biociências, Universidade de São Paulo. Matão Street, 277, 05508-090 São Paulo, SP Brazil

**Keywords:** Hydrolases, Fungal biology

## Abstract

β-glucosidases catalyze the hydrolysis β-1,4, β-1,3 and β-1,6 glucosidic linkages from non-reducing end of short chain oligosaccharides, alkyl and aryl β-D-glucosides and disaccharides. They catalyze the rate-limiting reaction in the conversion of cellobiose to glucose in the saccharification of cellulose for second-generation ethanol production, and due to this important role the search for glucose tolerant enzymes is of biochemical and biotechnological importance. In this study we characterize a family 3 glycosyl hydrolase (GH3) β-glucosidase (Bgl) produced by *Malbranchea pulchella* (*Mp*Bgl3) grown on cellobiose as the sole carbon source. Kinetic characterization revealed that the *Mp*Bgl3 was highly tolerant to glucose, which is in contrast to many Bgls that are completely inhibited by glucose. A 3D model of *Mp*Bgl3 was generated by molecular modeling and used for the evaluation of structural differences with a Bgl3 that is inhibited by glucose. Taken together, our results provide new clues to understand the glucose tolerance in GH3 β-glucosidases.

## Introduction

Under physiological conditions the β-glucosidases (Bgls - EC 3.2.1.21) catalyze the hydrolysis of β-1,4-glycosidic bonds at the non-reducing termini in alkyl- and aryl-β-D-glycosides, as well as in oligosaccharides containing 2*–*6 monosaccharides^1–4^. The Bgls are ubiquitous in nature and due to their wide range of substrate specificities, the Bgls play various biological roles^5^, and this diversity leads them to be considered as industrially important enzymes^6^.

In cellulolytic microorganisms, the Bgls may act as cellulolytic enzymes that synergistically function by converting cellulose to glucose. Together with endoglucanases (EC 3.2.1.4) and cellobiohydrolases (EC 3.2.1.91), β-glucosidases are involved in the degradation of cellulosic biomass^6–9^. Although the activities of cellobiohydrolases and endoglucanases are inhibited by the reaction product (cellobiose), Bgls can overcome this inhibition by the hydrolysis of cellobiose^10–12^. A deficiency in Bgls activity can result in the accumulation of cellobiose, leading not only to enzyme inhibition of the upstream enzymes, but also to the repression of enzyme biosynthesis, which results in limitations on hydrolysis yield^12–14^. Thus, Bgls can be considered to be the rate-limiting factor in the conversion of cellulose to glucose in biomass saccharification. Due to this important role, there is an increasing demand for the identification, production and characterization of new Bgls that retain their catalytic activity in the presence of glucose^15^.

*Malbranchea pulchella* is a thermophilic fungus found in fragments of decomposing plants or cellulose-containing material and is a good producer of trehalases^16^, xylanases^17,18^ and β-glucosidases^19^, and it may be considered promising for the production of enzymes of biotechnological interest. The objective of this study was the isolation of a glucose-tolerant GH3 β-glucosidase produced by *M. pulchella* together with the biochemical characterization and a structural study of this enzyme.

## Results

### MpBgl3 purification, identification and glycosylation analysis

**Figure 1:**
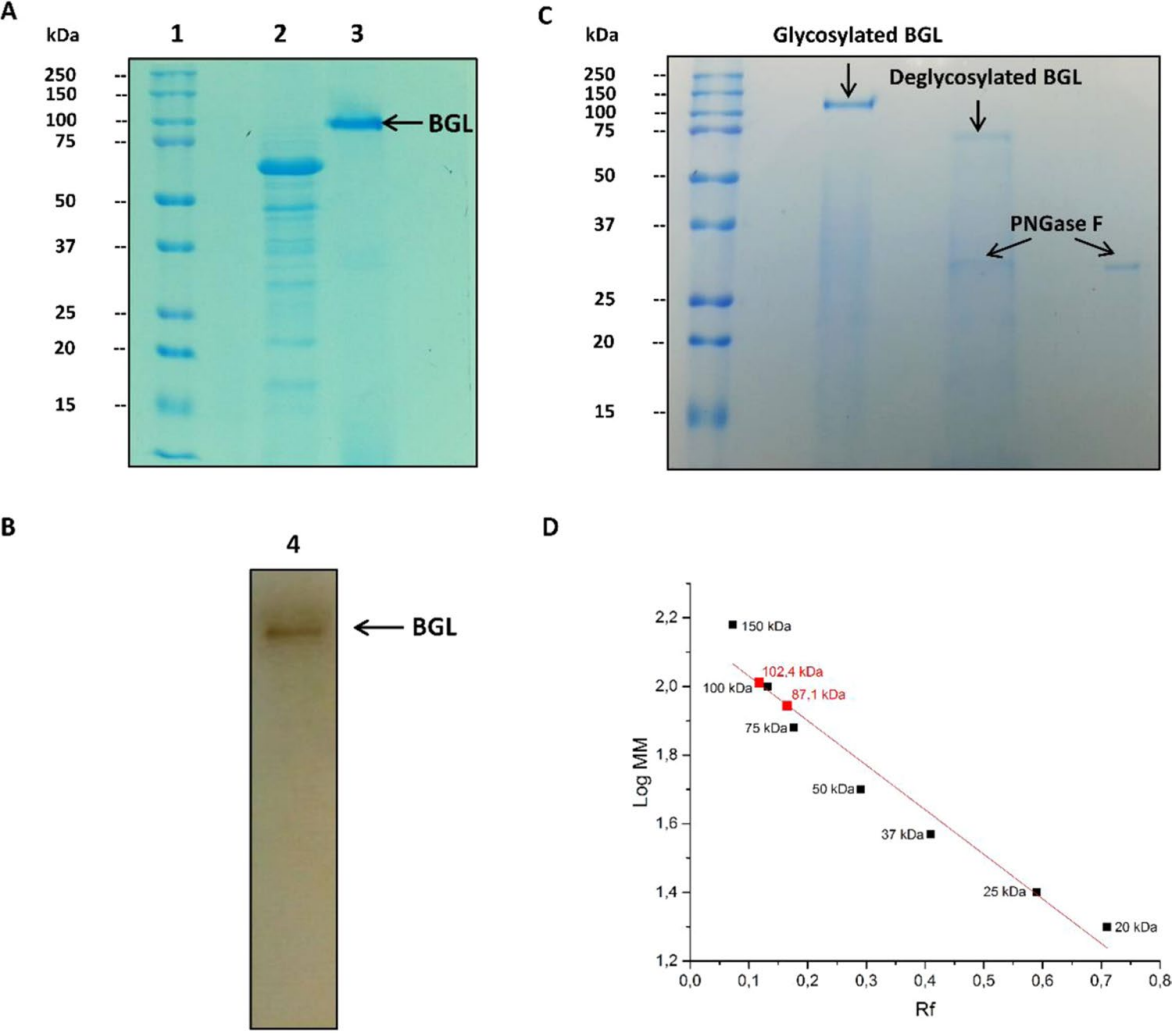
Purification, identification and deglycosylation effect of *M. pulchella* β-glucosidase (*Mp*Bgl3). (**A**) 10% SDS-PAGE stained with *Coomassie* Blue in (1) Dual Color Standards (BIO-RAD) molecular weight marker and (2) Crude extract produced by *M. pulchella* and (3) purified *Mp*Bgl3. (**B**) Zymogram in semi-denaturing conditions in gel 10% in (4) Dark band shows *Mp*Bgl3 activity. Effect of the deglycosylation on the *Mp*Bgl3 molecular weight. (**C**) Polyacrylamide gel electrophoresis 12% of the glycosylated and deglycosylated *Mp*Bgl3; (**D**) Determination of the molecular masses of glycosylated and deglycosylated *Mp*Bgl3 on SDS-PAGE. The band below the deglycosylated *Mp*Bgl3 is the PNGase F enzyme. Original photos of the gels can be found in Fig. S2.

The *Mp*Bgl3 was successfully purified by tangential ultrafiltration and elution using Fractogel DEAE resin, with a purification factor of 6.32, specific activity of 9.8 U/mg and an overall recovery of approximately 35% (Table 1). This purification protocol yielded a sample showing a single protein band with SDS-PAGE (Fig. 1A). In addition, the zymogram proved that the pure enzyme presented *Mp*Bgl3 activity (Fig. 1B). The identity of the purified protein was confirmed by mass spectrometry where the peptides HYILNEQEHFR and VNDFVNVQR from Af293 Bgl of *Aspergillus fumigatus*, GH3 family, were identified with a MASCOT score 90 (Table S1). These sequences were compared to a *M. pulchella* genome database (in collaboration with Dr. Rolf A. Prade of the Department of Microbiology and Molecular Genetics, University of Oklahoma), which identified the peptide HYILNEQEHFR within a GH3 family Bgls sequence of 90.34 kDa having a theoretical *pI* of 5.03. It was therefore concluded that the Bgl (*Mp*Bgl3) belongs to the GH3 family.

**Table 1:** Purification of the *M. pulchella* GH3 β-glucosidase.

Stage	Volume (mL)	Activity (U/mL)	Total activity (U)	Protein (mg/mL)	Specific activity (U/mg)	Purification factor	Recuperation (%)
Extracellular Crude extract	100	0.20	20.0	0.13	1.55	1	100
Tangential Ultrafiltration	30	0.51	15	0.09	5.62	3.63	75
DEAE-fractogel	14.4	0.49	7.05	0.05	9.80	6.32	35.28

Analysis of the pure *Mp*Bgl3 on SDS-PAGE (Fig. 1A) revealed that the enzyme has a molecular weight of approximately102 kDa and β-glucosidase activity was visualized by zymogram in semi-denaturing conditions (Fig. 1B). This is greater than the molecular weight calculated from the amino acid sequence (90.34 kDa), suggesting that the enzyme could be glycosylated. This question was addressed using a deglycosylation procedure combined with analysis of the profile of native and deglycosylated *Mp*Bgl3 by SDS-PAGE (Fig. 1C). The molecular weight of the deglycosylated *Mp*Bgl3 was estimated as 87.1 kDa (Fig. 1D). Thus, the glycosylation of the *Mp*Bgl3 corresponds to approximately 15% of the Bgl molecular weight.

### *Mp*Bgl3 kinetic constants, temperature and pH effect

**Figure 2:**
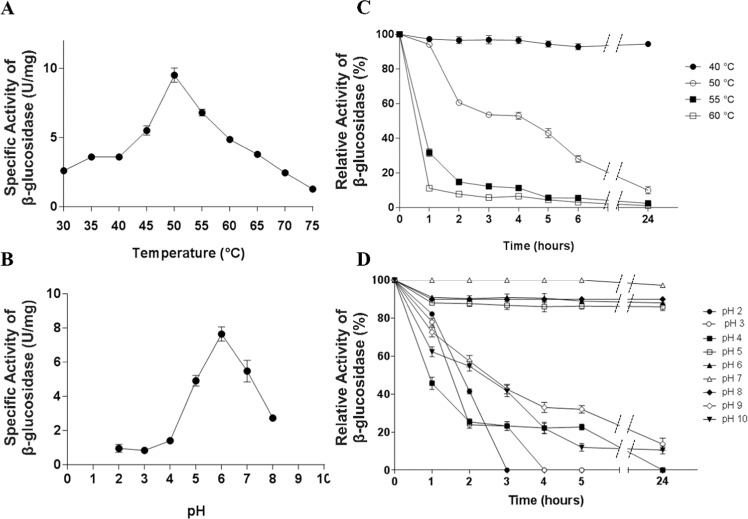
Effect of temperature and pH, and temperature and pH stability on *Mp*Bgl3 activity. (**A**) Temperature effect showing optimum temperature at 50 °C; (**B**) pH effect showing as optimum pH 6.0; (**C**) Thermal inactivation in which 100% activity was measured at t = 0. (**D**) pH stability where 100% activity was measured at t = 0, immediately before the addition of different buffers.

The kinetic parameters K_m_, V_max_ and K_cat_ of the *Mp*Bgl3 were evaluated by the SigrafW software. Purified *Mp*Bgl3 showed a K_m_ of 0.33 mM, V_max_ of 13.67 U/mg and K_cat_ of 26.6 s^−1^. The effects of temperature on the activity and stability of the *Mp*Bgl3 were also analyzed. As shown in Fig. 2A,B, the maximum activity of the *Mp*Bgl3 was observed at 50 °C and pH 6.0. Although the *Mp*Bgl3 was stable at 40 °C, and approximately 50% of its initial activity was retained after 4 hours in 50 °C. However, the enzyme lost all its activity after 4 hours at 55 and 60 °C (Fig. 2C). At the temperature of 50 °C, the optimal pH for the activity of the purified *Mp*Bgl3 was 6.0, and more than 85% of its maximum activity was retained in the pH range of 5.0 to 8.0 for up to 24 hours (Fig. 2D). In addition, under acidic conditions (pH 2.0–4.0), the enzyme retained approximately 80% of the initial activity for 1 h, however, the residual activity decreased during incubation over longer time scales. At pH 9.0 and 10.0, the enzyme retained 72% and 62% of the initial activity, respectively, for up to 1 hour, and thereafter showed a decreasing activity over longer times (Fig. 2D).

### Glucose effect

**Figure 3:**
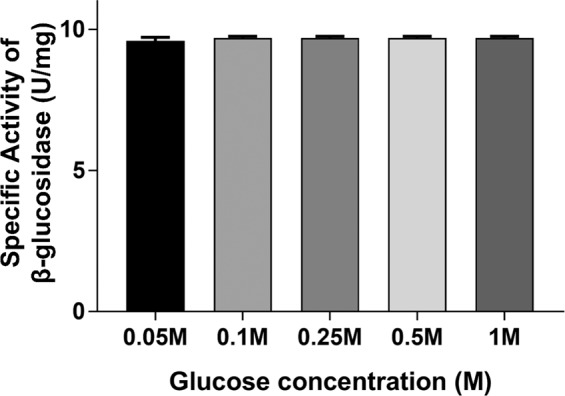
Glucose effect on the *Mp*Bgl3 activity. The glucose concentrations tested were 0.05 M; 0.1 M; 0.25 M; 0.5 M and 1 M. As control, the activity was measured without glucose, and in this case the specific activity was 9.8 U/mg.

β-glucosidases are usually inhibited by glucose and are a rate-limiting factor during enzymatic hydrolysis of lignocellulosic materials. Therefore, industrial applications, such as hydrolysis and fermentation, require Bgls that maintain activity in the presence of sugars as glucose. Evaluation of the activity of the *Mp*Bgl3 in the presence of glucose revealed that the enzyme displays high glucose tolerance (Fig. 3).

### Influence of metal ions and β-mercaptoethanol

**Figure 4:**
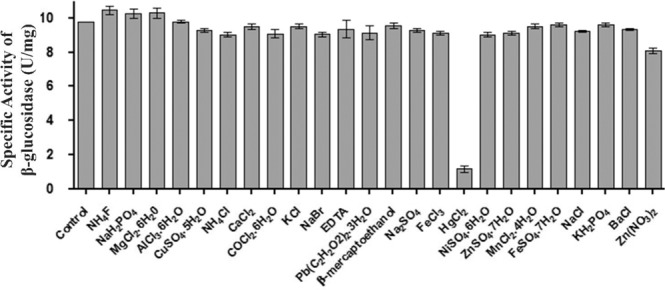
Influence of ions, EDTA and β-mercaptoethanol on *Mp*Bgl3 activity. The final concentration of each compound tested was 10 mM. The control sample was the activity measured in the absence of the compounds, represented in the graph as Bgl and therefore, equivalent to 9.8 U/mg. For this experiment the enzyme was previously dialyzed.

The effects of metal ions and reducing agents on enzymatic activity of the *Mp*Bgl3 are presented in Fig. 4. Significant inactivation was observed only in presence of HgCl_2_, and no significant effects were observed for any of the other substances tested. The lack of effect with EDTA suggests that the *Mp*Bgl3 does not require a metal ion cofactor for activity.

### Circular dichroism and molecular modelling of *Mp*Bgl3

**Figure 5:**
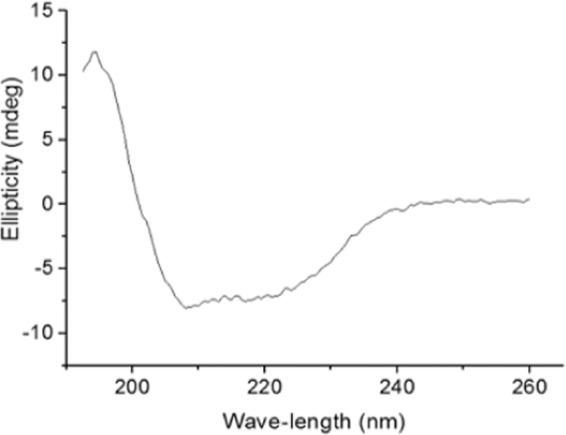
Far-UV circular dichroism spectrum of the purified *Mp*Bgl3.

The secondary structure of the purified *Mp*Bgl3 was analyzed by Far-UV CD spectrum (Fig. 5). The spectrum presents a positive peak at 194 nm and two negative peaks at 207 and 222 nm. These are characteristic of proteins that contain both α-helix and β-sheets, and deconvolution of the CD spectrum yields an estimate of 25% α-helix structure and 20% β-sheet structure.

The protein modelling C-score value of 0.81 for *Mp*Bgl3 indicates the reliability of the predicted tridimensional structure. With the aim of improving the quality of the model, energy minimization was performed using the Chiron server, which reduced the clash ratios in the modelled structure from 0.027 to 0.018 (Fig. S1). The overall stereochemical quality of the predicted tridimensional structure of *Mp*Bgl3 was evaluated using PROCHECK and Verify3D programs available at SAVE (The Structure Analysis and Verification Server) platform are presented in Table S2. The SAVE analyses showed that the majority of residues are in the favored region of Ramachandran plot and have a similar 3D/1D profile as comparing to well-characterized protein structures. These results suggest the modelled protein is reliable. According to the DSSP analysis, the *Mp*Bgl3 model presents 21.4% α-helix structure, 14.2% β-sheet (total of anti-parallel and parallel) structure, and 64.4% random coil elements which are in accordance with the CD analysis.

### The structural basis for glucose tolerance of the *Mp*Bgl3

**Figure 6:**
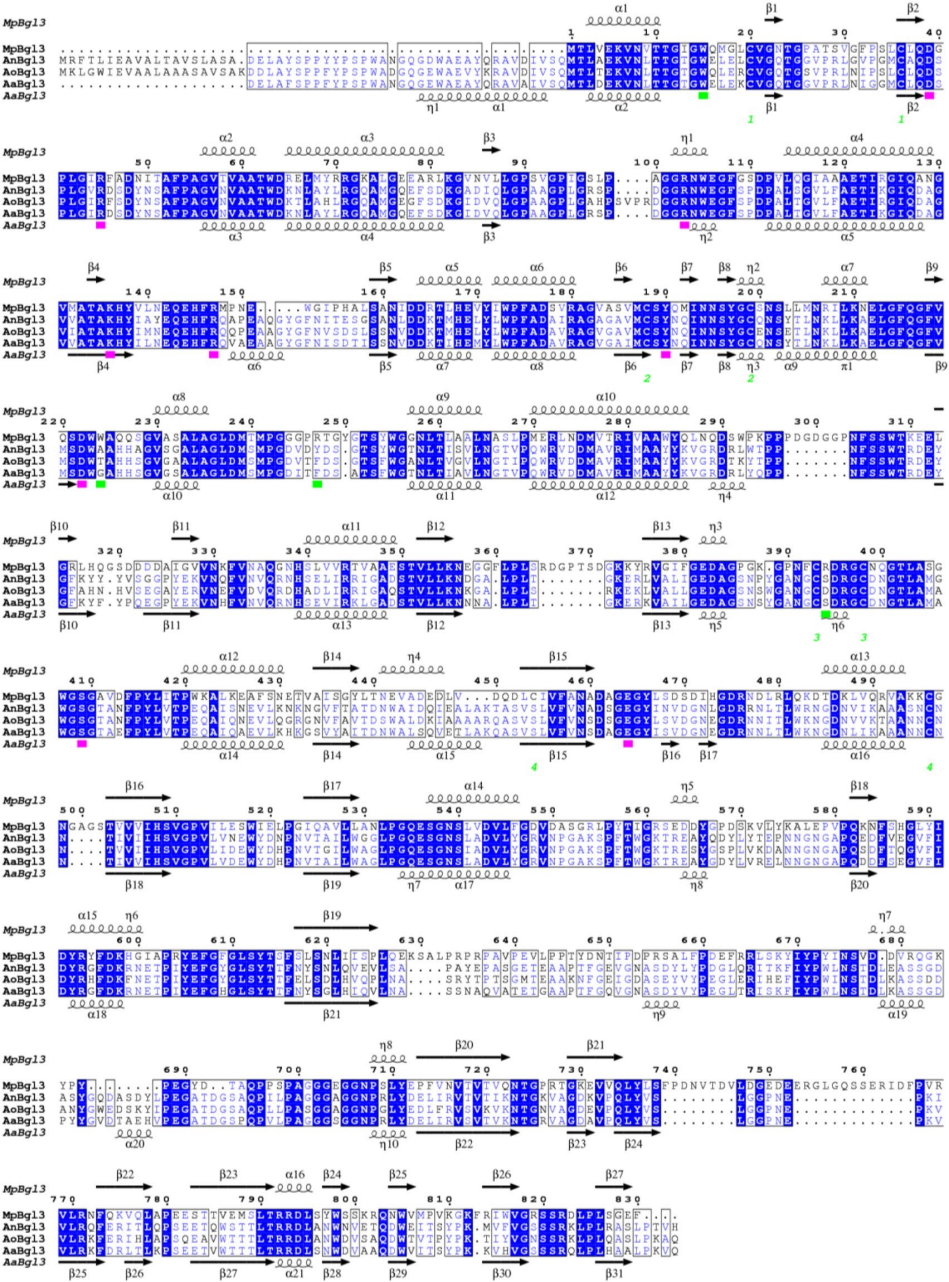
Sequence alignment of GH3 family β-glucosidases. *Mp*Bgl3 (*M. pulchella* Bgl3), *An*Bgl3 (*A. niger*), *Ao*Bgl3 (*A. oryzae* Bgl3), *Aa*Bgl3 (*A. aculeatus* Bgl3). The secondary structures of the *Mp*Bgl3 model and *Aa*Bgl3 crystal structure are shown above and below the amino acid sequences, respectively. The residues conserved between the sequences are show in the blue boxes. The green numbers correspond to the Cys residues that participate in the disulfide bridge in *Mp*Bgl3. Residues that directly bind the glucose to the glycone-binding site are indicated by pink squares. Residues around the aglycone-binding site are indicated by green squares. The alignment was performed using MULTALIN^48^ and graphically displayed using ESPript^49^.

To investigate the structural basis for glucose tolerance in Bgl3 enzymes, the amino acid sequence of the *Mp*Bgl3 was aligned with the glucose intolerant GH3 β-glucosidase from *A. niger* (*An*Bgl3; 46.1% sequence identity), *A. oryzae* (*Ao*Bgl3; 46.6% sequence identity), and *A. aculeatus* (*Aa*Bgl3; 47.2% sequence identity) (Fig. 6)^20,21^. For further analysis, the crystal structure of the glucose intolerant *Aa*Bgl3 (PDB entry 4IIG, K_I_ for glucose = 3.7 mM^20,22^ was superimposed on the modelled structure of the glucose tolerant *Mp*Bgl3with a rmsd of 1.02 Å (over 751 aligned Ca atoms).

**Figure 7:**
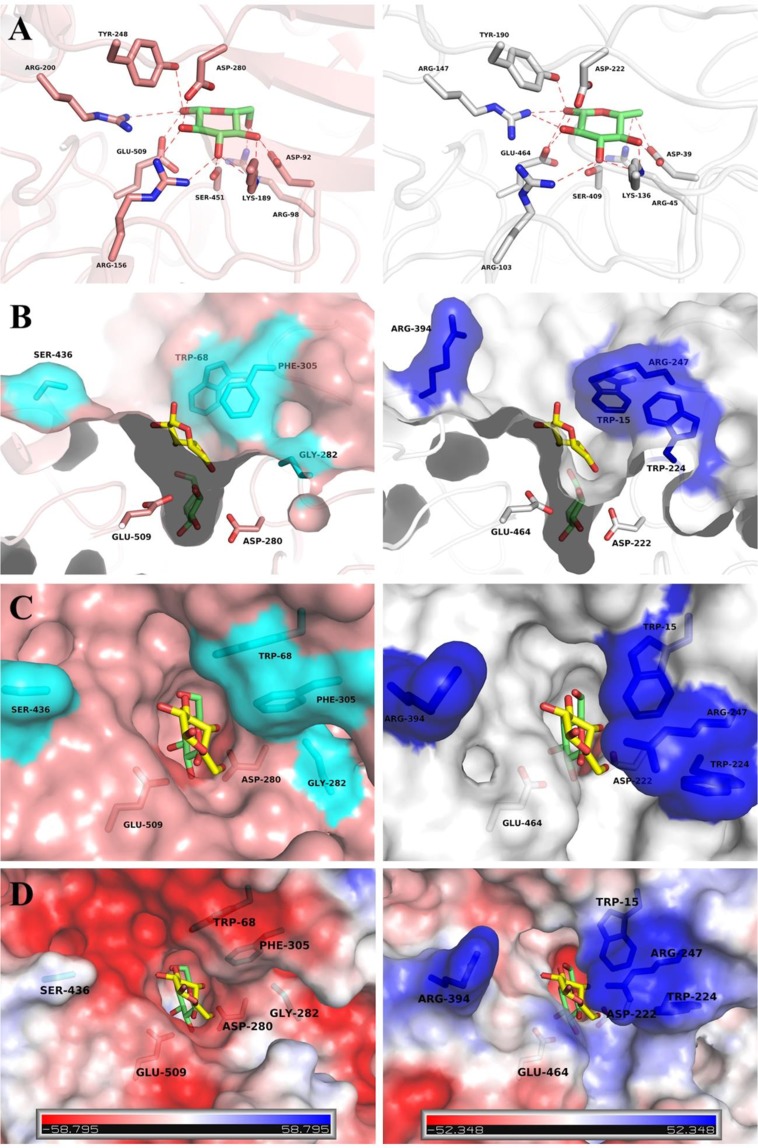
Structural comparison of *Mp*Bgl3 and *Aa*Bgl3. The *Mp*Bgl3 and AaBgl3 structures are represented on the right (in gray) and on the left (in pink) sides, respectively. The glucose at the glycone and aglycone-binding sites are represented in green and yellow, respectively. (**A**) Representation of the residues involved in the glucose interaction at the glycone-binding site. (**B**) Depth depiction of both, the catalytic site of the glucose tolerant (*Mp*Bgl3) and glucose intolerant (*Aa*Bgl3) enzymes. Residues that differ between the two enzymes and that contribute to the difference in the shape of the aglycone-binding site are represented in cyan and blue for *Aa*Bgl3 and *Mp*Bgl3, respectively. (**C**) View of the active site entrance, illustrating the wider entrance to the substrate binding cleft for *Aa*Bgl3 (inhibited by glucose) and the narrower entrance for the *Mp*Bgl3 (glucose tolerant). (**D**) Electrostatic potential surface of *Mp*Bgl3 and AaBgl3 generated by Pymol program, highlighting the differences on electrostatic environment of the catalytic site entrance. The images were generated with Pymol program (W.L. Delano, The PyMol Molecular Graphics System, DeLano Scientific).

The modelled 3D-structure and sequence alignments suggests that the *Mp*Bgl3 conserves the catalytic retaining mechanism that is typical of GH3 enzymes, where the glycone-binding site is fully conserved, together with the Asp222 and Glu464 residues which act as the nucleophile and the acid/base, respectively (Figs. 6 and 7A). Although the glycone-binding site is completely conserved, comparisons of the aglycone-binding site identified significant differences, which may be responsible for the change in the topology and electrostatic properties of the entrance to the catalytic site (Fig. 7B,C). It has been previously suggested that changes in the shape and the electrostatic properties of the aglycone-binding site were responsible for modulating the glucose tolerance of the GH1 β-glucosidases^23^. It was further suggested that GH1 Bgls are more glucose tolerant than GH3 Bgl3 because of the deeper catalytic cavity, a less accessible catalytic site entrance, and a reduced negatively charged patch in the aglycone-binding site that decreases the access of glucose in the glycone-binding site, resulting in an enzyme that is more tolerant to glucose^23^.

Comparative analysis of the *Mp*Bgl3 and *Aa*Bgl3 catalytic sites indicated that the shape and electrostatic properties of the deep active-site entrance is associated with GH3 Bgl glucose tolerance similarly as observed for GH1 family enzymes. The depth of the catalytic cleft in the *Aa*Bgl3 is greater than for the *Mp*Bgl3 (Fig. 7B). Furthermore, the Arg247 residue in the *Mp*Bgl3 is replaced by Phe305 in the AaBgl3 (Fig. 6 and 7B,C), and in the other GH3 Bgls that are inhibited by glucose this position is also occupied by aromatic residues (Phe in *Ao*Bgl3 and Tyr in *An*Bgl3), demonstrating that this difference may be important in modulating glucose tolerance (Fig. 6). In addition, the Trp224 residue in *Mp*Bgl3 is replaced by residues with less bulky side chains in the GH3 Bgl3 that are sensitive to glucose, such as Thr, Ala and Gly (Gly282 in AaBgl3) (Fig. 6). As shown in Fig. 7B,C, the presence of the Trp224 side chain in the *Mp*Bgl3 contributes not only to the restriction of the catalytic site entrance, but also pushes the Arg247 toward Trp15, and by changing the orientation of Trp15 further contributes to the narrowing of the entrance to the active site. Although the Trp15 residue in *Mp*Bgl3 is highly conserved in the different GH3 Bgls, the substitution of an aromatic residue at position 305 in glucose intolerant GH3 Bgl3 (for example Phe305 in *Aa*Bgl3) by Arg247 in *Mp*Bgl3 contributes to the loss of the aromatic stacking interaction with Trp15, perhaps facilitating the narrowing of the catalytic site entrance. Finally, the double substitution of residues Arg247 and Arg394 in the *Mp*Bgl3 by residues Phe305 and Ser436 in the AaBgl3 (Tyr and Ser in *An*Bgl3; Phe and Asp in *Ao*Bgl3), respectively, induce important changes on the active site entrance, introducing a more basic charge in the aglycone-binding site (Fig. 7D). Taken together, these results suggest that glucose tolerance by GH3 Bgl3 can be modulated by the depth, space and electrostatic characteristics of the catalytic site entrance in an analogous manner to the GH1 enzymes.

## Discussion

β-glucosidases are important enzymes that catalyze the rate-limiting reaction in the conversion of cellobiose to glucose in the saccharification of cellulose for second-generation ethanol production. Due to this important role, many groups around the world are focusing on the field of Bgls for several biochemical and biotechnological applications, aiming the optimization in bioreactor production, the reuse through enzymatic immobilization, improvement in activity by site-direct mutagenesis and others. The search for glucose tolerant enzymes has a great importance for the sugar and alcohol industry. In this study we standardized the production of Bgls with a buffered medium (Lummy medium^19^), that improved the Bgl3 physical-chemistry characteristics, a glucose hyper tolerant Bgl3 from a thermophilic fungus *Malbranchea pulchella* (*Mp*Bgl3) was purified and characterized.

The *Mp*Bgl3 was produced using 1% (w/v) cellobiose as carbon source in Lummy medium. This culture medium was chosen for being simple, buffered at pH 6.0 and to preferentially induce Bgl production. The purification performed by tangential ultrafiltration and ion exchange chromatography in DEAE-fractogel proved to be a fast, simple and an efficient protocol. Many Bgls have been purified using similar strategies^24–26^ and the purity of *Mp*Bgl3 was confirmed by SDS-PAGE, zymogram and by mass spectrometry analyses.

It is interesting to note that when evaluated for metal ions influence *Mp*Bgl3 did not present any significant activation and for this reason, *Mp*Bgl3 is not a metalloprotein. In contrast, mercury chloride could reduce 90% of *Mp*Bgl3 activity, and the majority of Bgls show a reduced total or partial activity in the presence of Hg^+2^. The relative effectiveness of the heavy metal ions as inhibitors has been reported to decrease the activity in the following order: Hg^+2^ > Ag^+^ >Cu^+2^ > Ni^+2^ > Cd^+2^ > Zn^+2^ > Co^+2^ > Fe^+3^ > Pb^+2^ > Mn^+2^ ^27^, and from all these heavy metals only the Hg^+2^ was able to inactivate the *Mp*Bgl3. It is know that the Hg^2+^ can inhibit the enzymatic activity acting on thiol sites present in the enzyme active site^28^, or by acting on R groups at the enzyme surface by changing the 3D structure and consequently its activity^28^. In the case of *Mp*Bgl3 it can be explained by model structure that shows Cys amino acids forming a disulfide bridge (Fig. 6), and it could be contributing to this result. The effect of ions on other Bgls is quite varied. For example, *Aureobasidium pullulans* Bgl retained its activity in the presence of all ions tested^15^, on the other hand, *Sporidiobolus pararoseus* Bgl was inhibited only by Ag^+2^ and Hg^+2^, and partially inhibited by Cu^+2^ and Zn^+2^ ^29^. Finally, *Penicillium pinophilum* Bgl was inhibited by Cu^+2^ and Pb^+2^ ^30^, and although the majority of metal ions do not inhibit Bgls activity, inhibition by Ag^+^, Hg^+2^, Cu^+2^ and Fe^+3^ has been frequently reported^31,32^.

The kinetic parameters of the *Mp*Bgl3 were determined, with a K_m_ of 0.33 mM, V_max_ of 13.67 U/mg and K_cat_ of 26.6 s^−1^, and they were compared to those of other Bgls. These enzymes from different organisms present significant differences in size and kinetic parameters, *i.e*. two Bgls were reported from *A. oryzae*, one with molecular weight (MW) of 130 kDa, K_m_ of 0.75 mM, V_max_ of 456 U/mg and K_cat_ of 651 s^−1^. The other had a MW of 100 kDa, K_m_ of 0.48 mM, V_max_ of 264 U/mg and K_cat_ correspondent to 373 s^−1^, using *p*NPG as substrate^24^. The *A. niger* Bgl showed MW of 95 kDa, K_m_ of 8 mM and V_max_ of 166 U/mg, for the same substrate^33^. Finally, a Bgl of *Trichoderma koningiopsis* FCD3-1 showed MW of 100 kDa, K_m_ of 1.21 mM, V_max_ of 314 U/mg and K_cat_ of 523 s^−1^ ^34^. Based on the literature values, it can be said that the *Mp*Bgl3 presents an affinity for the *p*NPG that is comparable to the Bgls from other fungi, but with a lower K_cat_.

The optimum *Mp*Bgl3 activity was estimated at 50 °C and pH 6.0, and it was similar to the Bgls from different organisms. Bgls usually exhibit optimal temperatures in the range of 40 °C to 60 °C and optimum pH in the range of 4.0 to 6.0 (Table 2)^15,24,29,35^. The *Mp*Bgl3 is a versatile enzyme that can be used and applied for several proposes since it is thermostable at 40 °C, but it also retained considerable activity for 4 hours at 50 °C, and in a pH range from 5.0 to 8.0 for 24 hours. The pH and temperature stability of Bgls may vary from one organism to another, but several authors have reported that Bgls for thermophilic fungi are stable at pH values ranging from 4.0 to 6.0 and at temperatures from 40 to 60 °C^15,24,29,35^.

**Table 2:** Properties of *M. pulchella* GH3 β-glucosidase.

Bgl3 organism	Glucose effect	Optimum pH	Optimum Temperature (°C)	pI	Molecular weight kDa	Reference
***Malbranchea pulchella***	No inhibition, 1 M	6	50	5.03	102	*This study*
***Agrobacterium tumefaciens***	*K*_i(GLC)_ (mM) = 558	7	52	—	—	^50^
***Aspergillus aculeatus***	*K*_i(GLC)_ (mM) = 3.7 +/− 0.1	5	65	4.7	93	^20^
***Aspergillus foetidus***	*K*_i(GLC)_ (mM) = 8.1 +/− 0.3	4.8	65	4.2	—	^20^
***Aspergillus japonicus***	*K*_i(GLC)_ (mM) = 9.2 +/− 0.1	—	65	4.7	121	^20^
***Aspergillus niger***	*K*_i(GLC)_ (mM) = 3.4 +/− 0.3	4.8	65	4.2	130	^20^
***Aspergillus tubingensis***	*K*_i(GLC)_ (mM) = 1.3 +/− 0.3	4.6	65	4.2	111	^20^
***Aspergillus oryzae***	*K*_i(GLC)_ (mM) = 2.9 ± 0.1	5	60	4.9	—	^51^
***Aspergillus fumigatus***	*K*_i(GLC)_ (mM) = 3.5	5	—	—	130	^52^
***Bacillus subtilis***	1 M, 70% residual activity	5	50	—	—	^53^
***Paenibacillus sp****.*	76% inhibition at 1.5 mM	6–7	37	—	50	^54^
***Penicillium decumbens***	110 mM, more than 90%	7	65	—	115	^55^
***Thermothelomyces thermophilus***	10 mM, 80% inhibition, competitive	5.4	65	—	—	^56^
***Trichoderma reesei***	competitive inhibition	5.5	—	—	—	^57^

The *Mp*Bgl3 showed hypertolerance to glucose concentrations of up to 1 M, which is an impressive result comparing to others Bgl3s from other fungi. Decker *et al*.^20^ characterized and calculated glucose Ki from different *Aspergillus* strains. In this work, it was possible to observe how unusual Bgls can be with high glucose tolerance, since *A. aculeatus* presented a K*i* of 3.7 +/− 0.1 (mM), *A. japonicus* 9.2 +/− 0.1 (mM*), A. foetidus* 8.1 +/− 0.3 (mM), *A. niger* 3.4 +/− 0.3 (mM) and *A. tubingensis* 1.3 +/− 0.3 (mM). Besides that, Zhu, *et al*.^21^ showed that just 4 g/L of glucose was enough to strongly inhibit the Bgls activities from *A. oryzae* and *A. niger*. In the present work it was not possible to calculate *Mp*Bgl3 Ki because the glucose concentration values at which the enzyme was tested did not inhibit it.

Another objective of this work was to study the structural basis of this effect by modelling of the 3D structure. A 3D structural model for the *Mp*Bgl3 was calculated based on the amino acid sequence similarity with the glucose intolerant *An*Bgl3 from *A. niger* (PDB entry 4IIG). Modelling in the presence of glucose inferred that the active site region of the *Mp*Bgl3 as well as the amino acids are important in the interaction with glucose. Previous studies suggested that changes in the shape and the electrostatic properties of the aglycone-binding site were responsible for modulating the glucose tolerance for Bgl1^23^. It was already published that Bgl1 used to present greater glucose tolerance then Bgl3 due to the deeper catalytic cavity and less accessible catalytic site entrance, reducing the negatively charged patch in the aglycone-binding site that decreases the access of glucose^23^. In this work, comparative analysis showed that although *Mp*Bgl3 was a Bgl3 it presented the shape and electrostatic properties of the deep active-site entrance similar as observed for Bgl1 enzymes. In other words, the finds of this work suggested that glucose tolerance by *Mp*Bgl3 could be modulated by the depth, space and electrostatic characteristics of the catalytic site entrance in an analogous manner to the GH1 enzymes. These results represent a new perspective for those working on the improvement of enzyme cloning and expression, or those working with site-directed mutagenesis as a perspective to improve β-glucosidase performance.

In conclusion, the present study reports the purification, biochemical, kinetic characterization and 3D-modelling of a ß-glucosidase GH3 (*Mp*Bgl3) from the thermophilic fungus *M. pulchella*. The hyperglucose tolerance of the *Mp*Bgl3 is of interest in industrial applications since glucose tolerant Bgls are not inhibited by feedback. When included in an enzyme cocktail for biomass saccharification, these tolerant enzymes may improve the hydrolysis efficiency by shifting the equilibrium towards product formation. Further work is currently in progress in order to evaluate the role of glucose-tolerant Bgls on biomass hydrolysis.

## Methods

### Production of *Mp*Bgl3

*M. pulchella* strain used in this study is deposited at the Ribeirão Preto Filamentous Fungi Collection of, at the Laboratory of Microbiology and Cell Biology, Department of Biology from the Faculty of Philosophy, Sciences and Letters of Ribeirão Preto, São Paulo, Brazil (FFCLRP). The fungus was maintained at 40 °C in Emerson medium^36^ for 7 days to propagate mycelial growth. A volume of 1.0 mL (final concentration of 10^6^ spores) of a conidial suspension of *M. pulchella* was inoculated into 125 mL Erlenmeyer flasks containing 25 mL of liquid Lummy medium (composed by: 0.4% yeast extract, 0.9% Na_2_HPO_4_, 0.05% MgSO_4_ and 0.35% citric acid)^19^, with cellobiose (1%) as the only carbon source. The cultures were incubated in an orbital shaker (180 rpm) for 72 h at 40 °C. The mycelia were subsequently, separated from the liquid medium by vacuum filtration on Whatman filter paper number 1, and the crude filtrate was used as the source of extracellular *Mp*Bgl3.

### Purification of *Mp*Bgl3 secreted by *M. pulchella*

The two-step purification of *Mp*Bgl3 was performed at 4 °C, in which 100 mL of the crude enzyme extract was concentrated and fractionated by tangential filtration using a Vivaspin™ 20 membrane (50 and 100 kDa cutoff, GE Healthcare Life Sciences, Uppsala, Uppland, SE). In this step the proteins greater than 50 kDa and smaller than 100 kDa were recovered in a total volume of 10 mL. the pH of this recovered fraction was adjusted to 7.0 with 25 mM Tris-HCl buffer pH 7.0, and loaded onto a Fractogel® EMD DEAE(M) (Merck Millipore Corporation, Darmstadt, Hessen, DE) (3 ×1 cm) column previously equilibrated with the same Tris-HCl buffer (25 mM, pH 7.0). The protein was eluted with a linear gradient of 0 to 1 M sodium chloride, and the fractions with Bgl activity were pooled and used in all subsequent experiments. The enzymatic extract and purified *Mp*Bgl3 were analyzed by SDS-PAGE 12%, stained with *Coomassie Blue*, and the protein concentration was estimated by the Bradford method^37^.

### Zymogram for *Mp*Bgl3

The zymogram was performed by semi-denaturing gel electrophoresis, with a sample buffer containing 0.002% bromophenol blue in 0.12 M Tris, pH 6.75 the sample was not boiled. Electrophoresis was performed at 4 °C in running buffer 0.025 M Tris-HCl, 0.19 M glycine and 0.1% SDS pH 8.3 at 30 mA and 120 V. After electrophoresis, the gel was washed in 0.2 M sodium acetate buffer, pH 5.0, for 10 min at room temperature and then incubated in 0.2 M sodium acetate buffer containing 0.1% (w/v) esculin (Sigma-Aldrich) and 0.03% (w/v) FeCl_3_ at 50 °C until the appearance of dark bands corresponding to *Mp*Bgl3 activity were observed^38^.

### Mass spectrometry

The *Mp*Bgl3 band in the 12% gel SDS-PAGE was cut and digested with 0.5 μg trypsin (Promega, Madison, WI, USA) in 17 μL of 0.1 M ammonium bicarbonate buffer pH 8.0. After digestion, the peptides were purified with Poros 50 R2 (PerSeptive Biosystems, Framingham, Massachusetts, USA) reverse phase column. The purified peptides were hydrated in 6 μL of a matrix solution (5 mg/mL α-ciano-4-hidroxycinnaminic acid in 50% acetonitrile and 0.1% trifluoroacetic acid (v/v)) and 2 μL of the hydrated sample were applied to the MALDI-TOF/TOF plate (Axima Performace, Kratos-Shimadzu, Manchester, UK). The MS/MS resulting spectra were analyzed using the MASCOT software (Matrix Science, London, UK) and the NCBInr/fungi database.

### Circular dichroism (CD)

The secondary structure of *Mp*Bgl3 was analyzed with a Jasco 810 spectropolarimeter (JASCO Inc., Tokyo, Japan) at wavelengths between 190–250 nm (far UVCD). The protein sample was diluted in sodium phosphate buffer pH 7.0, 10 mM to a concentration of approximately 0.1 mg/mL. Readings were performed in a quartz cuvette with an optical path length of 0.1 mm and the data collection used a scanning speed of 100 nm/min, spectral bandwidth of 1 nm, and response time of 0.5 s. Buffer spectra without protein were subtracted in all experiments, and the CD spectra of 9 accumulations were averaged. The measurement was performed at 25°C.

### Glycosylation analysis

Glycosylation of the *Mp*Bgl3 was analyzed using the endoglucanase PNGase F (Sigma-Aldrich Saint Louis, Missouri, USA). Approximately 5 µg of the *Mp*Bgl3, 5 µL of 5X reaction buffer and 1.25 µL of denaturing solution were mixed and incubated for 5 min at 100 °C. After cooling at room temperature 1.25 µL of Triton X-100 solution and 0.5 µL of the endoglucanase PNGase F were added and further incubated at 37 °C for 3 h. The product of this reaction was analyzed by electrophoresis on SDS-PAGE 12% and stained with *Coomassie Blue*.

### Measurement of BGL activity

The enzymatic activity was determined by *p*-nitrophenyl-β-D-glucopyranoside (*p*NPG) hydrolysis. The assay was initiated with 15 µL of enzyme 0.2 U/mL added to 10 µL of McIlvaine buffer pH 6.0 and 25 µL of *p*NPG (4 mM in H_2_O), and incubated for 5 min at 50 °C. The assay was stopped by adding 50 µL of 0.2 M Na_2_CO_3_ solution and free *p*-nitrophenol concentration was measured by the absorbance at 405 nm. The enzymatic unit (U) was defined as the amount of enzyme required to hydrolyze one micromol of substrate. The specific activity was defined as the number of units per mg of protein in the enzyme extract (U/mg).

### Influence of temperature and pH on *Mp*Bgl3 activity

In order to characterize the effect of temperature on *Mp*Bgl3 catalytic activity, *p*NPG hydrolysis was measured over the temperature range 30 °C to 75 °C using 15 μL of enzyme 0.2 U/mL, 10 μL of McIlvaine pH 6.0 and 25 μL of 4 mM *p*NPG. To evaluate the influence of pH on enzyme activity, the hydrolysis of *p*NPG was carried out under the same conditions varying the pH of the Mcllvaine buffer from 2.0 to 8.0, and maintaining a constant temperature of 50 °C.

The thermal stability of the *Mp*Bgl3 was evaluated at temperatures of 40 °C, 50 °C, 55 °C and 60 °C. In these experiments, the enzyme was incubated without the substrate and aliquots were withdrawn at predetermined times for enzyme assay. The pH stability of the purified enzyme free of substrate was evaluated at 25°C, at pre-defined incubation times, in a pH range varying from 2 to 10, using different buffers. After the incubation period, the enzyme activity was assayed as described above. The buffers used were: McIlvaine (pH 2–8), 50 mM glycine (pH 9–10). The results were expressed in Residual Activity (%), where the 100% value was the enzymatic activity before incubation.

### Kinetic characterization of *Mp*Bgl3

Determination of the kinetic parameters (V_max_ and K_m_) of *p*NPG hydrolysis by the purified *Mp*Bgl3 were determined in McIlvaine buffer pH 6.0 and 50 °C, and values of V_max_ and K_m_ were estimated using the SigrafW software^39^.

### Influence of ionic compounds, EDTA and β-mercaptoethanol

The inhibitory effect on the activity of *Mp*Bgl3 of various metal ions (as the salts NH_4_F, NaH_2_PO_4_, MgCl_2_.6H_2_O, AlCl_3_.6H_2_O, CuSO_4_.5H_2_O, NH_4_Cl, CaCl_2_, COCl_2_.6H_2_O, KCl, NaBr, Na_2_SO_4_, FeCl_3_, HgCl_2_, NiSO_4_.6H_2_O, ZnSO_4_.7H_2_O, MnCl_2_.4H_2_O, FeSO_4_.7H_2_O, NaCl, KH_2_PO_4_, BaCl, Zn(NO_3_)_2_), EDTA and β-mercaptoethanol was evaluated. The final concentration of each tested compound in the enzymatic reaction was 10 mM. Control sample was taken as the assay in the absence of any of the compounds tested. In these experiments the enzyme was previously dialyzed against distilled water.

### Glucose effect

To evaluate the effect of glucose on the *Mp*Bgl3 activity, the assay described at *Measurement of BGL activity* was performed in the presence of different glucose concentrations. The final concentrations of glucose tested for the pure *Mp*Bgl3 were 0.05 M, 0.1 M, 0.25 M, 0.5 M and 1 M at optimum pH and temperature. All experimental activities were expressed relative to the 100% activity measured without the addition of glucose.

### *Mp*Bgl3 modeling

The modelling of the *M. pulchella* GH3 three-dimensional structure was performed using the I-TASSER server^40–42^. The best model was selected based on I-TASSER C-score values. Energy minimization of the selected tridimensional model was performed using Chiron server^43^. The evaluation of the three-dimensional model was performed using the PROCHECK^44^ and Verify3D^45,46^ programs via the SAVES (The Structure Analysis and Verification Server) platform. The 2Struc (The Secondary Structure Server) platform was used to calculate the secondary structure composition of the *M. pulchella* GH3 model using the DSSP algorithm^47^.

### Ethical approval

The authors declare that no experiments on humans or animals were performed for this article.

## Supplementary information

Supplementary information
