# Supplementary material for: A Highly Glucose Tolerant ß-Glucosidase from Malbranchea pulchella (MpBg3) Enables Cellulose Saccharification
Source: Sci Rep. 2020 Apr 24;10:6998. doi: 10.1038/s41598-020-63972-y (PMC7181827; doi:10.1038/s41598-020-63972-y)
Supplement: Supplementary file 1 — Supplementary information [file 41598_2020_63972_MOESM1_ESM.docx]

**Supplementary Information**

A Highly Glucose Tolerant ß-Glucosidase from *Malbranchea pulchella* (*Mp*Bg3) Enables Cellulose Saccharification

Lummy Maria Oliveira Monteiro^a^, Ana Claudia Vici^b^, Matheus Pinto Pinheiro^c^, Paulo Ricardo Heinen^a^, Arthur Henrique Cavalcante de Oliveira^b^, Richard John Ward^b^, Rolf Alexander Prade^d^, Marcos S. Buckeridge^e^, Maria de Lourdes Teixeira de Moraes Polizeli^a,b^ *

*Table S1.* Amino acid sequence of the GH3 β-glucosidase from *Aspergillus fumigatus* Af293. The peptides corresponding to those identified by mass spectrometry are shown in bold. The sequences of these peptides were used for protein identification with the MASCOT software (Matrix Science, London, UK) and the NCBInr/fungi database.

| >gi\|70990956\|ref\|XP_750327.1\| beta-glucosidase [*Aspergillus fumigatus* Af293] |
| --- |
| *MRFGWLEVAALTAASVANAQVFDNSHGNNQELAFSPPFYPSPWADGQGEWADAHRRAVEIVSQMTLAEKVNLTTGTGWEMDRCVGQTGSVPRLGINWGLCGQDSPLGIRFSDLNSAFPAGTNVAATWDKTLAYLRGKAMGEEFNDKGVDILLGPAAGPLGKYPDGGRIWEGFSPDPALTGVLFAETIKGIQDAGVIATAK**HYILNEQEHFR**QVGEAQGYGYNITETISSNVDDKTMHELYLWPFADAVRAGVGAVMCSYNQINNSYGCQNSQTLNKLLKAELGFQGFVMSDWSAHHSGVGAALAGLDMSMPGDISFDDGLSFWGTNLTVSVLNGTVPAWRVDDMAVRIMTAYYKVGRDRLRIPPNFSSWTRDEYGWEHSAVSEGAWTK**VNDFVNVQR**SHSQIIREIGAASTVLLKNTGALPLTGKEVKVGVLGEDAGSNPWGANGCPDRGCDNGTLAMAWGSGTANFPYLVTPEQAIQREVISNGGNVFAVTDNGALSQMADVASQSSVSLVFVNADSGEGFISVDGNEGDRKNLTLWKNGEAVIDTVVSHCNNTIVVIHSVGPVLIDRWYDNPNVTAIIWAGLPGQESGNSLVDVLYGRVNPSAKTPFTWGKTRESYGAPLLTEPNNGNGAPQDDFNEGVFIDYRHFDKRNETPIYEFGHGLSYTTFGYSHLRVQALNSSSSAYVPTSGETKPAPTYGEIGSAADYLYPEGLKRITKFIYPWLNSTDLEDSSDDPNYGWQDSEYIPEGARDGSPQPLLKAGGAPGGNPTLYQDLVRVSATITNTGNVAGYEVPQLYVSLGGPNEPRVVLRKFDRIFLAPGEQKVWTTTLNRRDLANWDVEAQDWVITKYPKKVHVGSSSRKLPLRAPLPRVY |

*Table S2.* Evaluation of tridimensional model of the *M. pulchella* GH3 β-glucosidase.

| **Protein** | **PROCHECK (%)*** | **Verify3D (%)**** |
| --- | --- | --- |
| *Mp*Bgl3 | 94.7 | 90.60 |

*Percentage of amino acids in allowed region of Ramachandran plot

** Percentage of amino acids with scored >= 0.2 in the 3D/1D profile

**
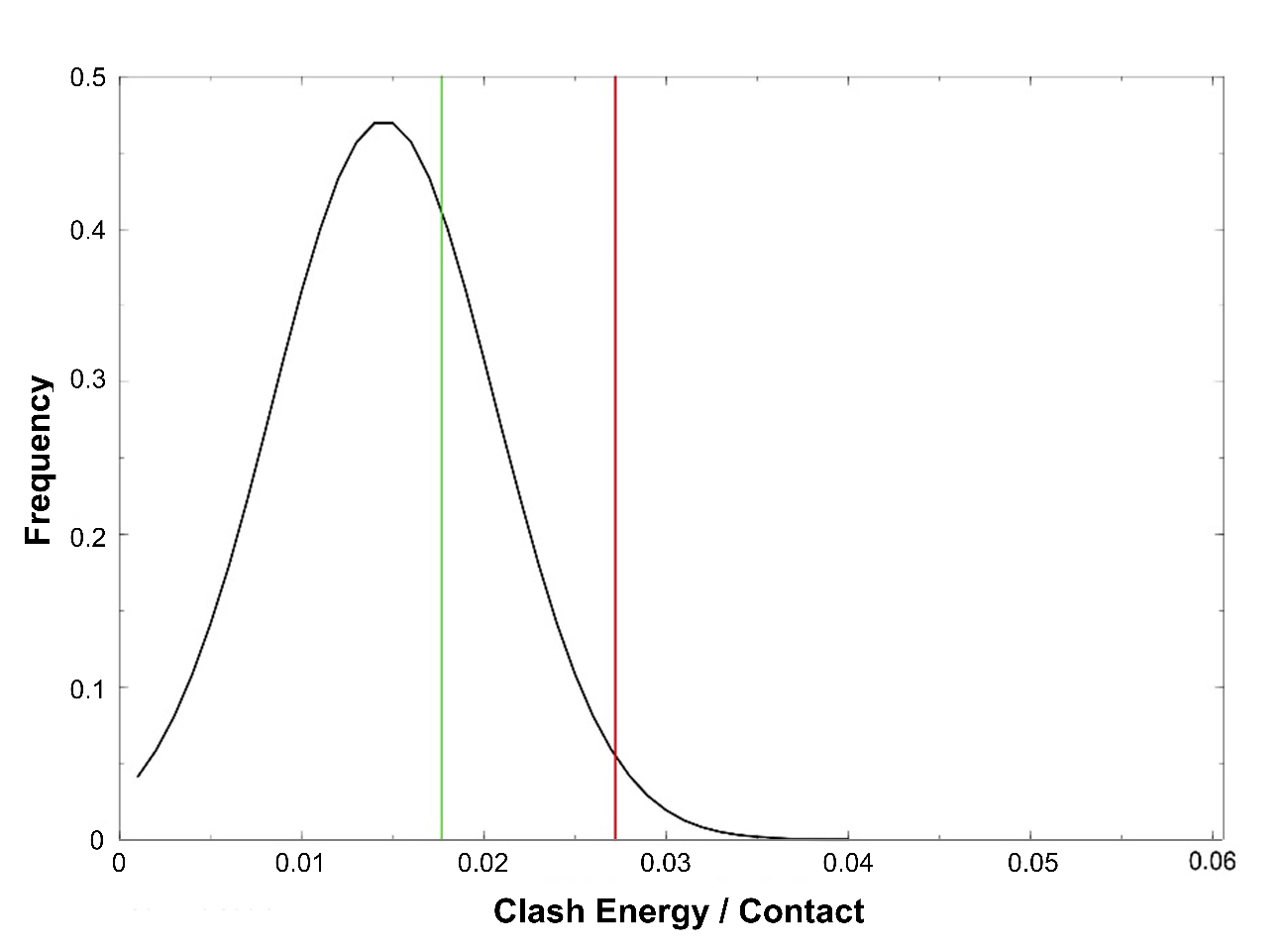
**

*Figure S1.* Normalized clash-score distribution plot of the Chiron server benchmark set indicating the initial and final clash-score of the MpBgl3 model. Benchmark Distribution (black); Initial Structure (red). Final Structure (green).


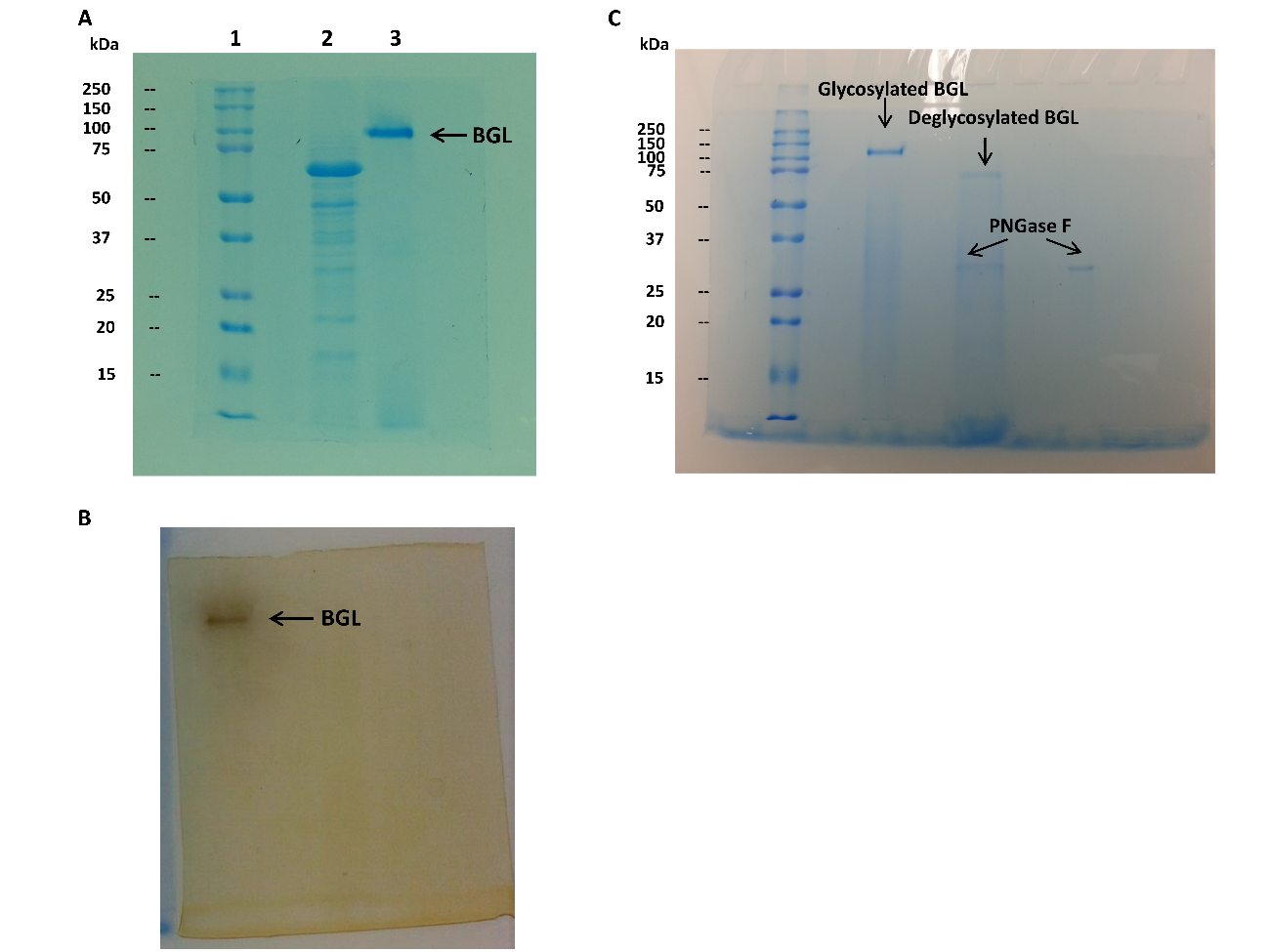


*Figure S2.* Original photos of the gels shown in Figure 1. (*A*) 10% SDS-PAGE stained with Coomassie Blue. (*B*) Zymogram in semi-denaturing conditions in 10% gel; (*C*) Polyacrylamide gel electrophoresis 12% of the glycosylated and deglycosylated MpBgl3. For more information see Figure 1.
